# Magnitude of malaria and associated factors among febrile adults in Siraro District Public Health facilities, West Arsi Zone, Oromia, Ethiopia 2022: a facility-based cross-sectional study

**DOI:** 10.1186/s12936-023-04697-x

**Published:** 2023-09-06

**Authors:** Yosef Yohanes Lakew, Anteneh Fikrie, Sisay Bedane Godana, Fatuma Wariyo, Wongelawit Seyoum

**Affiliations:** 1 Department of Nursing, Pharma College Hawassa Campus, P.O. Box 67, Hawassa, Ethiopia; 2 Departement of Public Health, Pharma College Hawassa Campus, P.O. Box 67, Hawassa, Ethiopia

**Keywords:** Malaria, Magnitude, Febrile adult patient, Siraro district, Oromia, West Arsi Zone

## Abstract

**Background:**

Despite significant efforts made to control malaria in Ethiopia, the disease remains one of the top public health problems in the country. Baseline malaria prevalence and associated factor at high malaria area is important to guide malaria control interventions, there was paucity of information regarding the study area. Therefore, the aim of this study was to determine prevalence of malaria and associated factors among febrile adults in Siraro district health facilities, West Arsi Zone, Oromia, Ethiopia.

**Methods:**

Institution-based cross-sectional study was conducted among 317 febrile adult patients at Siraro district health facilities. Structured pre-tested questionnaires were used to collect data. Epi-data version 3.1 and SPSS version 23 were used for data entry and analysis respectively. In order to identify factors associated with malaria infection bivariable and multivariable binary logistic regression analysis was employed, The Adjusted Odds Ratio (AOR) with a 95% confidence interval (CI) and p-value of < 0.05 was computed to show the strength of the association.

**Results:**

The overall prevalence of malaria at the study area was 130 (41.0%) [(95% CI 35.3–46.7)]. Occupation (being farmer) [(AOR = 6.05; 95% CI 1.38, 26.49)], having poor knowledge on malaria transmission [(AOR = 2.95 95%; CI 1.48–5.88)], house with wood wall [(AOR = 2.71; 95% CI 1.34–5.49)], and number of windows (≥ 3) in the house [(AOR = 6.82; 95% CI 1.05, 44.40)] were identified to be significantly associated with magnitude of malaria in the study area.

**Conclusion:**

The prevalence of malaria at the study area was high as compared with the national wide figures. Being farmer, having poor knowledge on malaria transmission, and housing condition (house with wood wall and houses with three and above windows) were found to be significantly associated with malaria infection in the study area. Therefore, there has to be an emphasis on addressing the factors by providing sustainable health education for the communities to improve their housing condition and knowledge of community on the way of malaria prevention.

## Background

Malaria is a life threatening, but easily preventable and treatable a vector-borne disease that continues to have a devastating impact on the health and livelihood of people around the world [1]. Malaria is caused by *Plasmodium* parasites, mainly *Plasmodium falciparum* and *Plasmodium vivax*, that are transmitted by the bite of infected female *Anopheles* mosquitoes [1]. *Plasmodium falciparum* is the most prevalent malaria parasite in the World Health Organization (WHO) African Region, accounting for 99.7% of cases, whereas *P. vivax* is the predominant parasite in the WHO Region of the Americas, representing 75% of malaria cases in 2018 [2]. Symptoms like fever, headache and chills usually appear 10–15 days after the infective mosquito bite and may be mild and difficult to recognize as malaria. However, if left untreated, *P. falciparum* malaria can progress to severe illness and death within a period of 24 h [3].

Malaria is one of the most severe public health problems worldwide and identified as a leading cause of death and disease in many developing countries [4]. Nearly half the world’s population lives in areas at risk of malaria transmission, in 87 countries and territories [3]. In areas with high transmission (sub-Saharan Africa) the most at risk population groups are: infants, children under 5 years of age, pregnant women, patients with HIV/AIDS, and people with low immunity moving to areas with intense malaria transmission [1]. In areas with lower transmission (such as Latin America and Asia), residents are less frequently infected. Many persons may reach adult age without having built protective immunity and are thus susceptible to the disease, including severe and fatal illness [4].

Globally in 2020, there were an estimated 241 million cases and 627,000 death of malaria [5]. This represents about 14 million more cases and 69,000 more deaths compared to 2019 [1, 5]. Approximately two-thirds of the additional deaths (47,000) were linked to disruptions in the provision of malaria prevention, diagnosis and treatment during the COVID-19 pandemic [3]. The WHO African Region, with an estimated 228 million cases in 2020, accounted for about 95% of cases [1, 4, 5]. In Africa, Nigeria (31.9%), the Democratic Republic of the Congo (13.2%), United Republic of Tanzania (4.1%) and Mozambique (3.8%) accounted for over half of all malaria deaths worldwide [1]. In Ethiopia, malaria has sustained to be one of the leading public health problems, it has found in nearly 70% of the country, where more than 60% the nations are at risk of the infection. In 2017/18, more than 1.2 million cases and 158 deaths had reported. Of the total (69.2%) cases were due to *P. falciparum* and 30.8% were P. *vivax* [6]. Malaria is also more than merely a tropical disease that claims a heavy toll on lives in sub-Saharan Africa (SSA) and is economically costly. The costs to individuals and their families include purchase of drugs for treating malaria at home; expenses for travel to, and treatment at, dispensaries and clinics; lost days of work; absence from school; expenses for preventive measures; and expenses for burial in case of deaths [4, 7]. Although malaria prevalence is occurs mostly in poor, tropical and subtropical areas of the world. Africa is the most affected due to a combination of different factors [4]. *Anopheles gambiae* complex, *Plasmodium falciparum*, local weather conditions often allow transmission to occur year round, scarce resources and socio-economic instability, parasite and vector control measures, and knowledge, attitudes and malaria prevention practices [8–13].

The global scale-up of malaria prevention interventions has saved millions of lives globally and cut malaria mortality by 36% from 2010 to 2020, leading to hopes and plans for elimination and ultimately eradication. However, the magnitude is still remains to be stagnant [5]. Moreover, the study area, Siraro district is one of high malarias district in West Arsi Zone, which has repeatedly affected by malaria epidemic (Source West Arsi zonal health department)*.* Even though the area categorized as high malarias area no study found about the magnitude and risk factor associated with malaria. Therefore, this study aimed to understand the burden and risk factors associated with malaria, which would help the government and stakeholders to plan strategies for malaria prevention and control.

## Methods

### Study design and setting

Institutional-based cross-sectional study design was conducted from June 25 to July 20/2022.

At public health facilities providing malaria treatment services at Siraro Woreda, Oromia Regional State, South Ethiopia. Siraro Woreda is located about 56 km to West of Shashamane city, capital of West Arsi Zone, and about 330 km from Addis Ababa capital city of Ethiopia. In the Woreda there are 6 rural clusters and 32 kebeles with total population of 207,541 (Male population = 99,619 and Female population = 107,921). Almost all areas of the Woreda are cash crop areas; the main crops cultivated in the Woreda are boloke and maize. A number of public and private health facilities are providing health care services for those populations. Public health facilities are one Primary Hospital, 6 health centres and 28 health posts. In addition to these, there are six private health facilities.

### Population, sample size determination and procedure

All febrile adults under the catchment area and randomly selected febrile adult patients who visited Siraro district public health facilities during the study period were the source and study population of our study. Sample size for the first objective was determined using a formula for a single population proportion **(n= (Zα/2)**^**2**^ *p (1 − p)/d^**2**^**)** by considering the following assumptions: prevalence of malaria in East Showa, Ethiopia, which was 25% [14], d = 5% marginal error and CI = 95% confidence interval (z = 1.96). In addition, a non- response rate was considered with 10% final sample size **=** 317.

Sample size for the second objective, which is risk factor malaria, is calculated using double population proportion formula taking more frequently observed associated factors for risk factor for malaria (age, outdoor sleeping and educational status) [12, 22] using Epi info became 94, 264, and 49, respectively. Finally, the larger sample size of 317 has selected, as it is the highest sample size to address both specific objectives of the study.

### Data collection tools and data quality control

The data were collect by a pretested structured interviewer-administered questionnaire, which were adapted from previous peer-reviewed literature [8–10, 13]. First, the questionnaire was prepared in English, and then translated to the regional working language “Afaan Oromo” by fluent speakers of both languages and then translated back to English to keep the consistency of the questionnaire. The questionnaire comprised four sections. The first section was Socio-demographic and economic characteristics such as age of mothers, religion, ethnicity, educational status, place of residence, marital status, occupational status, and income. Environmental factors: Presence of open and stagnant water around residency, history of infected by malaria, family size of the household, natural or manmade dam around residency, distance from the dam in km, roof and wall of house, and number of windows.

The participants’ level of knowledge were assess by eight items: (1) malaria is preventable, (2) stagnant water is mosquito breeding site, (3) mosquito biting time is at night, (4) female *Anopheles* mosquito transmit malaria, (5) feeling cold and rigor is sign of malaria, (6) fever is a sign of malaria, (7) malaria is curable disease, and (8) use of ITNs prevents malaria infection. Attitude of the participants was evaluated by eight items: (1) think anyone can get malaria; (2) malaria is a serious and life-threatening (fatal) disease, (3) the best way to prevent myself getting malaria is to avoid getting mosquito bites, (4) sleeping under a mosquito net during the night is one way to prevent myself getting malaria, (5) can be risky when malaria medicine is not taken properly and completely, (6) need to visit health centre/clinic when I am suspected of malaria, (7) think one can prevent him/herself from getting malaria, (8) malaria can be transmitted from person to person.

Practice of malaria prevention was evaluated by 10 questions about (1) How often do you visit the health center/clinic when you fall sick, (2) How do you describe your habit of visiting health centre/clinic when any other family member falls sick, (3) How often do you sleep in an insecticide-treated mosquito net (ITN), (4) How often do other family members sleep in mosquito nets, (5) How do you describe your habit of checking for holes/repair mosquito nets, (6) How often do you use anti-mosquito spray (IRS) in your house, (7) How do you describe your habit of trimming bushes around your home.

Three trained nurses and two BSc holders Health Officers working in Siraro district Health centres were participate as the data collectors and supervisors, respectively. Two days of training had bestowed for both data collectors and supervisors with regard to the importance of the study, how to keep privacy, and the confidentiality of the study participants. Moreover, 5% (n = 16) of questionnaires tested to nearby district (Shalla) health facility. Fortunately, there was no adjustment was done. The principal investigator together with supervisor checked and reviewed questionnaires on daily bases to ensure the completeness and consistency of information collected. The collected data coded, and entered using Epi-Data version 3.1 to maintain the quality of the data.

### Data processing and analysis

The Collected data entered by Epi-Data version 3.1 and exported to SPSS version 23 for data analysis. Descriptive and analytical statistical methods used to describe and infer the findings respectively. Tables, figures, and text used to present results. Binary logistic regression analyses run to assess the statistical association between malaria prevalence and each independent variable. Accordingly variables that was statistically significant at p-value of < 0.25 during bivariable binary logistic regression were entered into multivariable binary logistic regression to control the role of confounding variables. Multicollinearity was tested by using the variance inflation factor (< 10). A reliability analysis of the questionnaires was checked and Cronbach’s alpha showed the questionnaire were passed the acceptable reliability number (α = 0.87). The Hosmer–Lemeshow test used to assess the model fitness. Adjusted odds ratios (AOR) with 95% CI estimated to assess the strength of associations and statistical significance declared at a p-value < 0.05.

### Operational definition

#### Knowledge on malaria

Eight items used to assess the participants’ level of knowledge. Each item was assessed with a 2-point scale having a score of 0 = for each incorrect answer (No), and 1 = for each correct answer (Yes). Then the final score for each scale calculated by adding up the points obtained for the corresponding items by SPSS compute command. Accordingly, the overall score range from a minimum of 0 to a maximum of 8 points. Hence, those participants who answered above the mean score (≥ 5.44) regarded as having a good knowledge. Whereas participants who answered less than the mean score (< 5.44) of the questions were taken as having poor knowledge [11].

#### Attitude

To determine the attitude of the participants toward malaria, 8 items were used. Each item was assessed with a 5-point Likert scale with scores ranging 1–5 (1 = strongly disagree, 2 = disagree, 3 = undecided, 4 = agree, 5 = strongly agree). The overall score range from a minimum of 8 to a maximum of 40. Thus, participants who answered above the mean score (≥ 22.05) regarded as having a positive/favourable attitude. Whereas participants who answered less than the mean score (< 22.05) of the questions were taken as having negative/unfavorable attitude [11].

#### Practice

To determine the practice of the participants toward malaria prevention, seven items were used. Each item was assessed with a 3-point Likert scale with scores ranging 1–3 (1 = never, 2 = sometimes, 3 = always). The overall score range from a minimum of 7 to a maximum of 21. Thus, participants who answered above the mean score (≥ 12.35) regarded as having good practice. Whereas participants who answered less than the mean score (< 12.35) of the questions were taken as having poor practice [11].

## Results

### Socio demographic characteristics study participants

Three hundred seventeen (317) respondents were participated in the study with the response rate of 100%. The mean (± SD) age of the respondents was 34.73 (± 11.75) years. Majority of study subjects, 190 (59.94%) and 144 (45.43%) were females and Oromo ethnics respectively. Among the vast majority of respondents 220 69.4% of them were married. Above half of the study subjects 219 (69.1%) were rural resident. The majority educational status 135 (42.6%) was secondary followed by primary 89 (28.1%) (Table 1).

**Table 1:** Socio-demographic characteristics of febrile adult patients

Variables	Category	Frequencies	%
Sex	Male	127	40.06
	Female	190	59.94
Age	15–24 years	56	17.67
	25–34 years	127	40.06
	35–44 years	73	23.03
	≥ 45 years	61	19.24
Ethnicity	Oromo	144	45.43
	Amhara	96	30.28
	Hadiya	38	11.99
	Other	39	12.3
Religion	Orthodox	106	33.44
	Muslim	157	49.53
	Protestant	39	12.3
	Catholic	15	4.73
Marital status	Single	79	24.92
	Married	220	69.4
	Divorced	18	5.68
Residence	Rural	98	30.9
	Urban	219	69.1
Educational status	Primary	89	28.1
	Secondary	135	42.6
	Diploma and above	66	20.8
	No formal education	27	8.5
Occupational Status	House wife	101	31.9
	Driver	103	32.5
	Farmer	51	16.1
	Government employ	36	11.4
	Merchants	26	8.2
Income Eth. Birr	> 1500	20	6.31
	1500–2500	89	28.08
	≥ 2500	208	65.62
Family size	< 5	199	62.78
	≥ 5	118	37.22

### Environmental and housing condition of febrile adult patients

Of 317 study participants’ 72 (22.71%) houses were tacked (attached) and 157 (49.53%) houses wall were constructed from wood. Among 317 respondents 189 (59.62%) of them responded that there is open and stagnant water around their residency. Of 317 study subjects 95 (29.97%) of them has no ITNS in their house and from those ITNS ownership 71 (22.40%) of them has < 2 ITNS (Table 2).

**Table 2:** Environmental and housing condition of febrile adult patients in Siraro district health facilities, Oromia regional state Southern Ethiopia 2022

Variables	Category	Frequency	Percent (%)
Housing condition	Corrugated iron sheet	107	33.75
	Mud	138	43.53
	Tacked	72	22.71
Number of window of the house	Only one	163	51.42
	Two	136	42.9
	Three and above	18	5.68
Type of the wall of the house	Cement	145	45.74
	Wood	157	49.53
	Mud	15	4.73
Presence of a stagnant water around residency	Yes	189	59.62
	No	128	40.38
Presence of man-made or natural damp at your locality	Yes	17	5.36
	No	300	94.64
Attitude level	Un favorable attitude	176	55.52
	Favorable attitude	141	44.48
Availability of ITNS in the house	Yes	222	70.03
	No	95	29.97
Number of ITNS/household	> 2	70	31.53
	≤ 2	152	68.47
How often you sleep under ITNS	Always	119	37.54
	Sometimes	117	36.91
	Never	81	25.55

### Knowledge of study participants about malaria prevention measures

Among 317 respondents’ majorities 245 (77.3%) of them replied that; Malaria is preventable disease and 136 (42.9%) respond that; stagnant water is not mosquito breeding site. Of 317 study participants 228 (71.9%), 244 (77%) of them respond that; feeling cold/rigor and fever is sign of malaria respectively. The overall good knowledge status of the study participants was 162 (51%) (Table 3).

**Table 3:** Malaria related knowledge of febrile adult patients in Siraro district health facilities, Oromia regional state Southern Ethiopia 2022

Variables	Category	Frequency	Percent (%)
Malaria is preventable	Yes	245	77.3
	No	72	22.7
Stagnant water is mosquito breeding site	Yes	181	57.1
	No	136	42.9
Mosquito biting time at Night	Yes	214	67.5
	No	103	32.5
Female Anofilace Mosquito can transmit malaria	Yes	199	62.8
	No	118	37.2
Feeling cold and rigor is sign of malaria	Yes	228	71.9
	No	89	28.1
Fever is sign of malaria	Yes	244	77
	No	73	23
Malaria is curable disease	Yes	224	70.7
	No	93	29.3
ITNS prevents malaria infection	Yes	190	59.9
	No	127	40.1
Over all knowledge level	Poor knowledge	155	48.9
	Good knowledge	162	51.1

### Attitude of study participants about towards malaria

Of 317 febrile adult patients at Siraro district 82 (25.9%) and 69 (21.8%) of them disagree and strongly disagree, respectively, on malaria is a serious and life-threatening (fatal) disease. From a total study subjects about; 89 (28.1%) of them were agreed while 78 (24.6%) of them were disagreed on avoid mosquito breeding site (stagnant water) prevent malaria. Majorities of study participants 85 (26.8%) greed that sleeping under a mosquito net during the night is way of malaria prevention (Table 4).

**Table 4:** Malaria-related attitude of febrile patients in Siraro district health facilities, Oromia regional state, Southern Ethiopia, 2022

Variables	Category	Frequency	Percent (%)
Do you agree anyone can get malaria	Strongly disagree	103	32.5
	Disagree	93	29.3
	Undecided	57	18.0
	Agree	45	14.2
	Strongly agree	19	6.0
Do you agree malaria is a serious and life-threatening (fatal) disease	Strongly disagree	69	21.8
	Disagree	82	25.9
	Undecided	53	16.7
	Agree	74	23.3
	Strongly agree	39	12.3
Do you agree avoid mosquito breeding site (stagnant water) prevent malaria	Strongly disagree	60	18.9
	Disagree	78	24.6
	Undecided	46	14.5
	Agree	89	28.1
	Strongly agree	44	13.9
Do you agree sleeping under a mosquito net during the night is way of malaria prevention	Strongly disagree	71	22.4
	Disagree	73	23
	Undecided	45	14.2
	Agree	85	26.8
	Strongly agree	43	13.6
Do you agree it is risky when malaria medicine is not taken properly and	Strongly disagree	78	24.6
	Disagree	87	27.4
	Undecided	30	9.5
	Agree	78	24.6
	Strongly agree	44	13.9
Do you agree to visit health facility if someone suspected himself malaria	Strongly disagree	84	26.5
	Disagree	80	25.2
	Undecided	31	9.8
	Agree	68	21.5
	Strongly agree	54	17
Do you agree one can prevent him/herself from getting malaria	Strongly disagree	77	24.3
	Disagree	78	24.6
	Undecided	36	11.4
	Agree	70	22.1
	Strongly agree	56	17.7
Do you agree malaria transmitted from person to person	Strongly disagree	86	27.1
	Disagree	74	23.3
	Undecided	26	8.2
	Agree	84	26.5
	Strongly agree	47	14.8
Attitude	Un favourable attitude	176	55.52
	Favourable attitude	141	44.48

### Prevalence of malaria among febrile adult patients at Siraro district

**Fig. 1:**
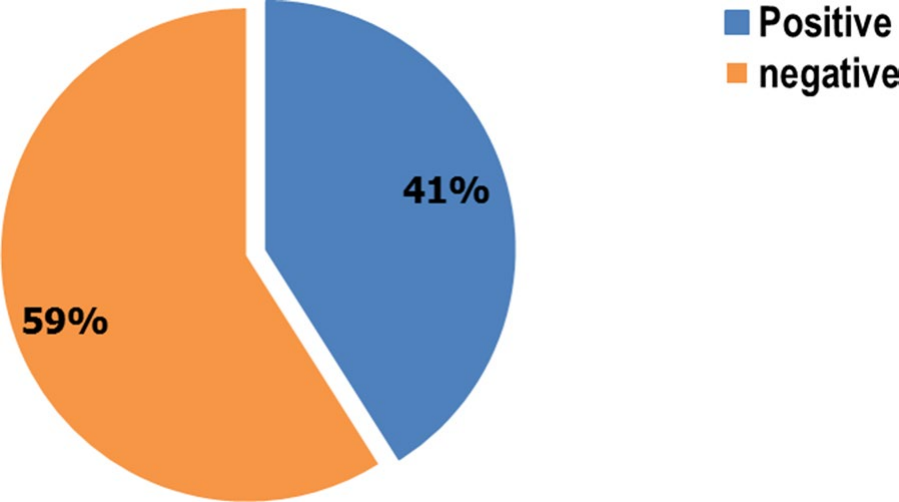
Test positivity for malaria among febrile adult patients. Prevalence of positive test for malaria among febrile adult patients in Siraro district health facilities, West Aris Zone, Oromia, Southern Ethiopia 2022”

The overall prevalence of malaria at the study area was 130 (41.0%) (95% CI 35.3–46.7) (Fig. 1).

### Factors associated with magnitude of malaria among febrile adult patients

In bi-variable binary logistic regression analysis from socio demographic factors; family size, occupational status, and educational status, ethnicity were nominated for multivariable binary logistic regression analysis. Likewise, knowledge, frequency of sleeping under ITNS, number of ITNS owner ship, housing condition, number of window available and construction of the wall were candidate variables multivariable analysis.

At the final multivariable model: Occupation (being farmer) [(AOR = 6.05; 95% CI 1.38, 26.49)], having poor knowledge on malaria transmission [(AOR = 2.95 95%; CI 1.48–5.88)], housing wall material (wood) [(AOR = 2.71; 95% CI 1.34–5.49)], and number of windows (≥ 3) [ (AOR = 6.82; 95% CI 1.05, 44.40)] were significantly associated with magnitude of malaria in the study area.

Accordingly, the odds of malaria prevalence were 6 times higher among famers as compared to merchants [(AOR = 6.05; 95% CI 1.38, 26.49)]. Participants who have poor knowledge on malaria transmission were nearly three times higher odds of contracting malaria as compared to those participants who have good knowledge [(AOR = 2.95 95%; CI 1.48–5.88)]. Participants whose house wall constructed form wood were nearly three times more likely to develop malaria as compared to participants whose housing wall constructed from cement [(AOR = 2.71; 95% CI 1.34–5.49)]. On the other hand, peoples living in the houses with three and above windows were 6.82 times (AOR = 6.82; 95% CI 1.05, 44.40) more likely infected by malaria as compared to peoples living with one window house (Table 5).

**Table 5:** Factors associated with malaria prevalence among febrile adult patients in Siraro district health facilities, Oromia regional state Southern Ethiopia 2022 (n = 317)

Variables	Category	Positive for malaria		COR	AOR
		Yes (%)	No (%)	95% CI	95% CI
Family size	< 5	73 (56.15)	126 (67.38)	1	1
	≥ 5	57 (43.85)	61 (32.62)	1.61 (1.01–2.56)	1.23 (0.55–2.76)
Occupational status	House wife	36 (27.69)	65 (34.76)	0.76 (0.31–1.81)	3.31 (0.85–12.87)
	Driver	37 (28.5)	66 (35.29)	0.76 (0.31–1.83)	2.22 (0.58–8.44)
	Farmer	30 (23.08)	21 (11.23)	1.95 (0.74–5.04)	6.05 (1.38 26.49)**
	Government employ	16 (12.31)	20 (10.70)	1.09 (0.39–3.02)	2.44 (0.54–11.07)
	Merchants	11 (8.46)	15 (8.02)	1	1
Knowledge	Poor knowledge	44 (33.85)	111 (59.36)	2.85 (1.79–4.54)	2.95 (1.48–5.88)**
	Good knowledge	86 (66.15)	76 (40.64)	1	1
Roof of the house	Corrugated iron sheet	32 (24.62)	75 (40.11)	1	1
	Mud	64 (49.23)	74 (39.57)	2.02 (1.19–3.45)	1.45 (0.61–3.46)
	Tached	34 (26.15)	38 (20.32)	2.09 (1.12,3.90)	1.06 (0.37–3.05)
House wall	Cement	50 (38.46)	103 (55.08)	1	1
	Wood	75 (57.69)	78 (41.71)	1.74 (1.09–276)	2.71 (1.34–5.49)**
	Mud	5 (3.85)	6 (3.21)	0.95 (0.30–2.93)	1.21 (0.28–5.21)
Number of window	Only one	60 (46.1)	103 (55.0)	1	1
	Two	58 (44.62)	78 (41.71)	1.28 (0.80–2.03)	1.08 (0.53–2.16)
	Three and above	12 (9.23)	6 (3.21)	3.43 (1.22–6.62)	6.82 (1.05–44.40)**

*AOR* adjust odds ratio, *CI* confidence interval

*Significant at *p <* 0.05

**Significant at p value < 0.001

## Discussion

The malaria National Malaria Strategic Plan (NMSP) for the years 2014–2020 had anticipated that by 2020 all households living in malaria endemic areas would have the knowledge, attitudes and practice towards malaria prevention and control, to reduce malaria cases by 75% from baseline of 2013 and to eliminate malaria in selected low transmission areas in Ethiopia [15]. Thus, this facility-based cross-sectional study assessed the prevalence of malaria and associated factors among febrile adults in Siraro District Public Health Facilities, Oromia Regional State, South West Ethiopia. Consequently, in this study a prevalence of malaria was 41.0% [(95% CI 35.3–46.7)]. Being farmer, participants with poor knowledge, housing wall material (wood), and numbers of windows (≥ 3) of the house were significantly associated with malaria.

The prevalence of malaria in this study is consistent with a systematic review and meta-analysis done in Ethiopia (37.6%) based on data obtained from studies conducted on mixed regions of low lands [16]. However, it is exceeding than studies done in East Shewa Zone of Oromia Regional State 25% [14], Benna Tsemay district, Southern Ethiopia 6.1% [8], and in migrant laborer’s in West Armachiho District, Northwest Ethiopia were 18.4% [17], Chagni Health centre, Northwest Ethiopia 7.3% [18], Shewa Robit, North central Ethiopia 19% [19], and Mizan-Aman Southwest Ethiopia [20]. The observed difference in the magnitude might be due to the difference of study area and period (change of climatic condition of the year) and in the implementation of the practice of malaria prevention and control measures at the community level. However, the result of this study is higher as compared with the previous studies report from Ashanti Region, Ghana 73% [21], Afar Region, Ethiopia 64% [9], and rural villages of mainland Equatorial Guinea 69% [22]. This gap might be due to the difference in geographical location and malaria endemic area. Moreover, it could be due to varied intense intervention implemented by the government health authorities of the region.

Agriculture and malaria has intertwined, a study found that irrigated rice-growing communities are exposed to more mosquitoes and higher malaria risk [23]. This study also found that, participants who are farmers were 6 times more likely of contracting malaria than merchants. This is consistent with the results of other studies conducted in West Armachiho District Northwest Ethiopia [17], Ndop District Hospital, Northwest Region of Cameroon [24], Northern Namibia [25], Kilosa district, Central Tanzania [26]. The similarities across the studies might be due to the nature of occupation of farmers related to engagement in frequent outdoor activities to their Agricultural Practice during malaria peak period of transmission. This finding contradicted with study done at settlement areas in the district of Juruena, Mato Grosso state in Brazil [27]. This inconsistence could be due to the difference in the living standard of the study participants.

Participants who have poor knowledge on malaria transmission methods were nearly three times more likely of being infecting with malaria as compared to participants who have good knowledge. The finding has supported by study done at southwestern coastal region of India [28], Malawi [29], South Africa [30], and Rwanda [31]. This is because people with good knowledge about malaria had more chance of engaging in malaria prevention practices [11]. Additionally, having a good knowledge on malaria transmission methods; leads to engage in different malaria prevention measures to prevent themselves from malaria infection.

In this study, the housing condition has found to be significantly associated with the magnitude of malaria infection among the study participants in Siraro district. Study participants who lives in the house with wood wall were more likely to have malaria infection than participants who were lives in the house with cement wall. Likewise, peoples living in the houses with more than three windows were higher likely of infecting by malaria as compared to peoples living with one window house. The finding is in line with study conducted in Eritrea where malaria infection is mostly common among peoples living in mud wall houses [32]. Similarly, a qualitative study done at rural Tanzania found that participants lives in houses with mud walls compared to plastered or brick walls, open eaves compared to closed eaves and unscreened windows compared to screened windows [33]. Evidence from the Nigeria malaria indicator survey revealed that the odds of malaria infection was significantly higher among participants with poor housing conditions [34]. This might be due to the reason that unimproved wall of the house makes conducive environment for resting and entrance of the mosquito. This implies that improving house condition has to be encouraged and emphasized as one of the preventive methods of attacking by the mosquito (helps as physical barrier between the host and the vectors).

## Conclusion

The prevalence of malaria at the study area was high as compared with the national wide figures. Factors like; occupational status being farmer, having poor knowledge on malaria transmission, housing condition (house with wood wall and houses with three and above windows) were found to be significantly associated with the observed magnitude of malaria in the study area. Therefore, there have to be given an emphasis on addressing the root causes of the problem via providing sustainable health education for the communities to improve their housing condition. Siraro district health office and health facilities in collaboration with stakeholders should have to work on malaria prevention strategy to reduce this high burden of malaria cases in the district.

## Data Availability

Data is not available for online access, however readers who wish to gain access to the data can write to the corresponding author Yosef Yohanes at abigiyayosef@gmail.com.

## Abbreviations

AOR: Adjusted odd ratio
CI: Confidence interval
HMIS: Health management information system
ITN: Insecticide treated net
RDT: Rapid diagnostic test
SPSS: Statically package for social science
USAID: United States of America international development
WHO: World Health Organization
